# Organ‐at‐risk dose prediction using a machine learning algorithm: Clinical validation and treatment planning benefit for lung SBRT

**DOI:** 10.1002/acm2.13609

**Published:** 2022-04-23

**Authors:** N. Patrik Brodin, Leslie Schulte, Christian Velten, William Martin, Sydney Shen, Jin Shen, Amar Basavatia, Nitin Ohri, Madhur K. Garg, Colin Carpenter, Wolfgang A. Tomé

**Affiliations:** ^1^ Institute for Onco‐Physics Albert Einstein College of Medicine Bronx NY USA; ^2^ Department of Radiation Oncology Montefiore Medical Center Bronx NY USA; ^3^ Siris Medical Burlingame CA USA; ^4^ Department of Radiation Oncology Massachusetts General Hospital Boston MA USA; ^5^ Department of Urology Montefiore Medical Center Bronx NY USA; ^6^ Department of Otorhinolaryngology‐Head & Neck Surgery Montefiore Medical Center Bronx NY USA; ^7^ Department of Neurology Albert Einstein College of Medicine Bronx NY USA

**Keywords:** dose prediction, machine learning, lung SBRT

## Abstract

**Objective:**

To quantify the clinical performance of a machine learning (ML) algorithm for organ‐at‐risk (OAR) dose prediction for lung stereotactic body radiation therapy (SBRT) and estimate the treatment planning benefit from having upfront access to these dose predictions.

**Methods:**

ML models were trained using multi‐center data consisting of 209 patients previously treated with lung SBRT. Two prescription levels were investigated, 50 Gy in five fractions and 54 Gy in three fractions. Models were generated using a gradient‐boosted regression tree algorithm using grid searching with fivefold cross‐validation. Twenty patients not included in the training set were used to test OAR dose prediction performance, ten for each prescription. We also performed blinded re‐planning based on OAR dose predictions but without access to clinically delivered plans. Differences between predicted and delivered doses were assessed by root‐mean square deviation (RMSD), and statistical differences between predicted, delivered, and re‐planned doses were evaluated with one‐way analysis of variance (ANOVA) tests.

**Results:**

ANOVA tests showed no significant differences between predicted, delivered, and replanned OAR doses (all *p* ≥ 0.36). The RMSD was 2.9, 3.9, 4.3, and 1.7Gy for max dose to the spinal cord, great vessels, heart, and trachea, respectively, for 50 Gy in five fractions. Average improvements of 1.0, 1.4, and 2.0 Gy were seen for spinal cord, esophagus, and trachea max doses in blinded replans compared to clinically delivered plans with 54 Gy in three fractions, and 1.8, 0.7, and 1.5 Gy, respectively, for the esophagus, heart and bronchus max doses with 50 Gy in five fractions. Target coverage was similar with an average PTV V100% of 94.7% for delivered plans compared to 97.3% for blinded re‐plans for 50 Gy in five fractions, and respectively 98.4% versus 99.2% for 54 Gy in three fractions.

**Conclusion:**

This study validated ML‐based OAR dose prediction for lung SBRT, showing potential for improved OAR dose sparing and more consistent plan quality using dose predictions for patient‐specific planning guidance.

## 1 INTRODUCTION

Radiation therapy treatment planning is an iterative process with the goal of achieving the best possible treatment plan for a given patient. A treatment plan is generated following the physician's prescription and is then discussed and further optimized until all clinical goals are met or an acceptable compromise has been achieved between target coverage and organ‐at‐risk (OAR) sparing. This can be a time‐consuming process, especially when dealing with complex intensity‐modulated radiation therapy (IMRT) treatments. Oftentimes the discussion regarding trade‐offs and achievable OAR sparing for an individual patient requires having access to a fully generated treatment plan, or set of plans if the treatment involves sequential boosts. Automatic plan generation and dose prediction using machine learning (ML) methods provide an excellent opportunity for streamlining the treatment planning workflow.^1^

Recently, substantial progress has been made in the field of knowledge‐based planning (KBP) as a way to automate the treatment planning process and assist treatment planners.^2^, ^3^, ^4^, ^5^, ^6^, ^7^, ^8^ Currently, the main goals of KBP strategies are to increase efficiency by reducing the time and effort needed to generate a clinically acceptable treatment plan and to reduce the inter‐ and intra‐planner variability in plan quality that is inherent to manual treatment plan generation, which has been shown previously using a set of prostate cancer cases.^4^ Head and neck cancer (HNC) is another site where KBP efforts have been focused, with one group successfully showing that KBP strategies developed at one institution can be implemented for patients at other institutions.^7^

Dose prediction is another avenue for ML methods to improve the radiation therapy treatment planning workflow, with HNC being a commonly investigated treatment site.^9^, ^10^, ^11^, ^12^ Dose prediction strategies strive to estimate patient‐specific OAR doses without the need to generate a treatment plan, facilitating quick and efficient discussions about achievable plan quality and trade‐offs to consider. A recent study evaluated two different methods of DVH prediction for the treatment of nasopharyngeal cancer and rectal cancer.^10^ The authors found that prediction of specific dose‐volume histogram (DVH) points was achievable within 5% accuracy for most of the studied OARs.

Institutions typically have OAR dose constraints or guidelines that need to be met for a treatment plan to be considered clinically acceptable. However, depending on patient anatomy and disease location there may be cases for which OAR dose constraints are met, even though the treatment plan is not optimal and further OAR sparing could be achieved.^13^ While KBP strategies have shown great promise for improving consistency and reducing variability in treatment plan quality, dose prediction offers a further avenue for pushing OAR sparing and plan quality beyond what has been previously achieved in the library of treatment plans used to generate a KBP model. Estimating the expected OAR doses based on the anatomy of an individual patient creates a set of patient‐specific dosimetric guidelines that could be used to achieve the best treatment plan, akin to the “best feasible DVH” estimation strategy previously reported.^9^ Furthermore, having access to dose predictions from different fractionation protocols would allow the treating physician to rapidly assess the most suitable option for a given patient without the need for comparative treatment plans.

Here we performed a study to assess the performance of an ML dose prediction algorithm for two different fractionation protocols for patients treated with lung stereotactic body radiation therapy (SBRT). We also evaluated the estimated benefit in terms of OAR sparing fromhaving access to these dose predictions upfront in the treatment planning process.

## 2 MATERIALS AND METHODS

### 2.1 Patient cohort

The training data used to build the dose prediction models consisted of an anonymized dataset of 209 lung cancer patients previously treated with SBRT, with 21.6% of the training data coming from our institution. In the training cohort, 121 patients were treated with 54 Gy in three fractions and 88 were treated with 50 Gy in five fractions. The test set consisted of 20 patients treated at our institution in 2020 that were not part of the training cohort. Ten of these patients were treated with 50 Gy in five fractions and the other ten with 54 Gy in three fractions. Eight patients were treated on a Varian TrueBeam linear accelerator, 11 on a TrueBeam Edge, and one patient on a Trilogy.

### 2.2 Machine learning model building

ML models were trained to estimate the OAR DVH metrics utilized in our institutional practice for lung SBRT (cf. Table 1), which are based on recommendations from RTOG 0618,^14^ RTOG 0813^15^ ^,^ and the AAPM TG‐101 report.^16^ For each OAR dose metric, a separate model was constructed to estimate OAR doses for SBRT prescriptions of 50 Gy in five fractions and 54 Gy in three fractions. Predictive models were generated using a gradient‐boosted regression tree algorithm, implemented using Python scikit‐learn v. 0.23.1. This type of model is a non‐parametric supervised learning algorithm with decision rules learned from the training data.^17^ Predictive features used for these models have been reported in detail previously^18^; they consisted of a variety of geometric calculations based on the planning CT scans and associated target and OAR contours, and historical data regarding prior treatment directives. To prevent overfitting, the hyperparameters of the ML algorithm (learning rate, loss function, number of estimators, tree depth, minimum number of samples per split, and number of boosting stages) were selected using grid searching with fivefold cross‐validated L3 (cubed) error on the training set. A detailed description of the model hyperparameters can be found in Table S1 of the Supporting Information. The L3 loss function was chosen for the hyperparameter grid search to increase the importance of minimizing max error of the training cohort and the authors direct the interested reader to Hastie et al.^19^ for a detailed discussion of the loss function.

**TABLE 1:** Treatment planning guidelines in terms of target coverage and organ‐at‐risk dose limits for the two lungs stereotactic body radiation therapy (SBRT) protocols studied

50 Gy in five fractions	54 Gy in three fractions
PTV V100% ≥ 95%	PTV V100% ≥95%
Spinal cord *D*_max_ < 30 Gy	Spinal cord *D*_max_ < 18 Gy
Spinal cord V13.5_Gy_ < 0.5 cm^3^	Spinal cord V13.5 _Gy_ < 0.5 cm^3^
Esophagus *D*_max_ < 52.5 Gy	Esophagus *D*_max_ < 27 Gy
Heart *D*_max_ < 52.5 Gy	Heart *D*_max_ < 30 Gy
Skin *D*_max_ < 32 Gy	Skin *D*_max_ < 24 Gy
Trachea *D*_max_ < 52.5 Gy	Trachea *D*_max_ < 30 Gy
Bronchus *D*_max_ < 52.5 Gy	Rib V30_Gy_ < 30 cm^3^
Great vessels *D*_max_ < 52.5 Gy	Whole lungs‐GTV V20_Gy_ < 10%
Rib V37.5_Gy_ < 30 cm^3^	
Whole lungs‐GTV V12.5_Gy_ < 1500 cm^3^	

### 2.3 Model performance testing

Dose predictions for the 20 test patients (10 per fractionation protocol) were obtained by feeding the planning CT scan along with target and OAR contours to the ML models implemented in the InsightRT CDS software v.3.1 (Siris Medical, Burlingame, CA, USA) as part of a research agreement with our institution. All planning CT scans were acquired with 1.25 mm slice thickness and the Eclipse AAA v.15.6 dose calculation model with 1.25 mm dose grid was used for all dose calculations. The prediction dose metrics were extracted and compared to the dose metrics from the DVHs of the clinically delivered treatment plans extracted from the Eclipse treatment planning system (Varian Medical Systems, Palo Alto, CA, USA) for the same 20 patients.

### 2.4 Blinded re‐planning comparison

To estimate the benefit of having access to these dose predictions in the treatment planning process, we performed a blinded re‐planning study. The CT scan and contour data were anonymized and uploaded to the Eclipse treatment planning system. Three junior planners were then tasked with generating treatment plans for these patients, without any access to the clinically delivered plans, but with access to the tabulated dose predictions from the ML models. Planners were instructed to meet that at least 95% of the target volume receives the prescription dose (PTV V100% ≥ 95%) and our institutional OAR dose constraints for lung SBRT in Table 1. The suggested treatment technique was volumetric‐modulated arc therapy (VMAT) with two or three arcs not entering through the contralateral lung, generating the plan on the same treatment machine as the clinical plan was delivered, ensuring the same collimation system and available beam geometry. Once completed, the plans were reviewed by a senior medical physicist making sure they met the standard for clinical acceptability, and the DVH dose metrics were tabulated and compared to the predictions and to those from the clinically delivered plans.

### 2.5 Statistical analysis

The DVH dose metrics from the predictions were compared to those from the clinically delivered plans by calculating the root‐mean square deviation (RMSD) for each dose metric. Hence, the RMSD quantifies the average uncertainty in the predicted dose compared to what was delivered clinically. We also assessed the RMSD between the replanned and clinically delivered plans. The mean difference for each OAR dose metric between the clinically delivered plans and the blinded re‐plans was tabulated, as well as the mean difference between the prediction estimates and the clinically delivered plans. Maximum doses (*D*_max_) were calculated as the highest dose to at least 0.03 cm^3^ of the corresponding OAR, following our institutional standard. To compare the OAR doses between all three scenarios (predicted, delivered, and replanned) we used one‐way analysis of variance (ANOVA) tests following assessment of normality and equal variance assumptions. All statistical analyses were performed using MATLAB R2018b (The MathWorks Inc, Natick, MA, USA), and *p* < 0.05 would be considered a statistically significant difference between the groups.

## 3 RESULTS

**FIGURE 1:**
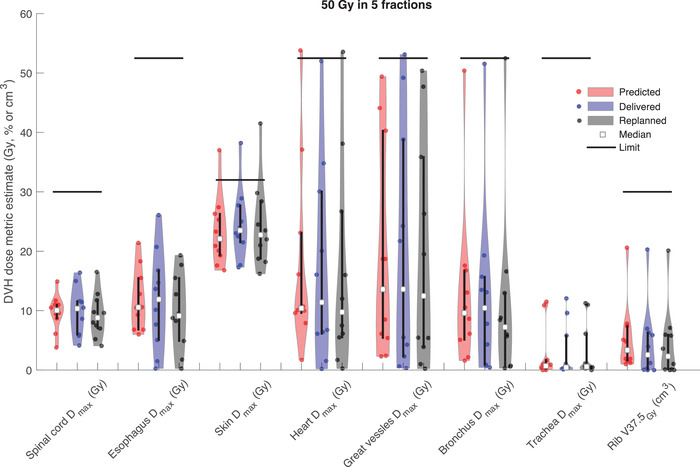
Violin plots showing the distribution of predicted, delivered, and replanned dose metrics for each organ‐at‐risk (OAR) for the 50 Gy in five fractions protocol. Vertical bars represent the inter‐quartile range and horizontal bars show the corresponding OAR tolerance limit

**FIGURE 2:**
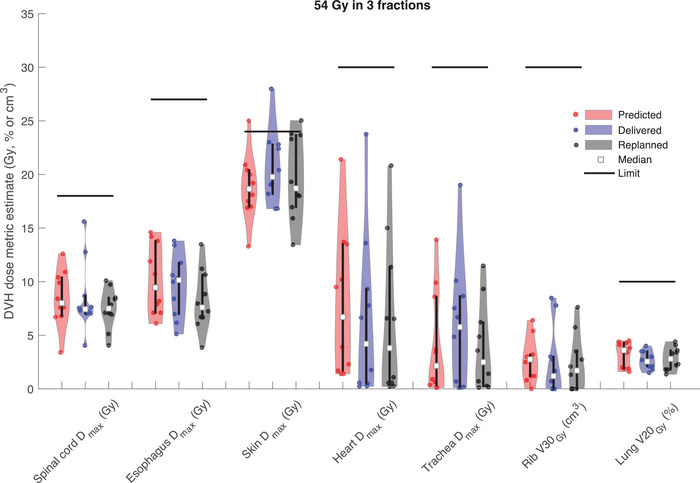
Violin plots showing the distribution of predicted, delivered, and replanned dose metrics for each organ‐at‐risk (OAR) for the 54 Gy in three fractions protocol. Vertical bars represent the inter‐quartile range and horizontal bars show the corresponding OAR tolerance limit

Comparisons of the DVH dose metrics between the predicted, delivered, and replanned scenarios are shown in Figure 1 for 50 Gy in five fractions and Figure 2 for 54 Gy in three fractions, evaluated on the test set of 20 patients. Figure 1 shows that the predicted max doses to the spinal cord, esophagus, heart, great vessels, bronchus, and trachea agreed well with the clinically delivered plans. In the blinded replans, the max doses to esophagus, heart, and bronchus having were on average 1.8, 0.7 ,and 1.5 Gy lower, respectively, compared to the clinically delivered plans. There were a few instances where the replanned doses to skin, heart, and bronchus were higher than for the clinically delivered plans, owing to the higher priority placed on target coverage in the blinded replan for those cases. Figure 2 shows that the predicted and delivered max doses for spinal cord and esophagus, and the volumetric dose measures for ribs and whole lungs minus gross tumor volume (GTV) agreed well also for the 54 Gy in three fractions protocol. For the skin and trachea max doses the model predictions were lower than those from the clinically delivered plans, which turned out to be achievable in the blinded replans with access to these model estimates. Improvements of on average 1.0, 1.4 ,and 2.0 Gy were seen for spinal cord, esophagus, and trachea max doses, respectively, in the blinded replans compared to the delivered plans.

Table 2 shows the quantitative results for the 20 test patients in terms of the RMSD estimates between the different scenarios and the mean difference for the various dose metrics. For both fractionation protocols, the ANOVA *p*‐values were ≥0.36 for all reported dose metrics, and as such there were no significant differences between the predicted, delivered, or replanned doses. The RMSD shows low average prediction uncertainty, at or below 10% of the corresponding dose constraint limit for all OARs except for esophagus *D*_max_ in the 50 Gy in five fractions protocol. The relative mean difference between predicted and delivered doses across all OAR DVH metrics was between 0.3% to 3.7% of the tolerance limit for 50 Gy in five fractions and 0.3% to 7.6% for 54 Gy in three fractions. The smallest differences were seen for the heart and esophagus max doses, and the largest difference was seen for the skin max dose. Target coverage was similar with an average PTV V100% of 94.7% for clinically delivered plans compared to an average PTV V100% of 97.3% for blinded re‐plans for 50 Gy in five fractions, and respectively 98.4% versus 99.2% for 54 Gy in three fractions.

**TABLE 2:** The dose prediction performance for each of the organs‐at‐risk included in the respective fractionation protocol, as estimated by the root‐mean square deviation and mean the difference between predicted and delivered doses, and replanned and delivered doses, as well as one‐way analysis of variance (ANOVA) comparisons between all three scenarios

	50 Gy in five fractions				
	RMSD (Predicted vs. Delivered)	Mean difference (predicted ‐ delivered)	RMSD (replanned vs. delivered)	Mean difference (replanned ‐ delivered)	ANOVA *p*‐value
Spinal cord *D*_max_	2.9 Gy	−0.3 Gy	2.2 Gy	−0.7 Gy	0.90
Esophagus *D*_max_	5.3 Gy	0.1 Gy	2.9 Gy	−1.8 Gy	0.78
Heart *D*_max_	4.3 Gy	0.6 Gy	2.4 Gy	−0.7 Gy	0.99
Skin *D*_max_	2.9 Gy	−1.2 Gy	2.2 Gy	−0.2 Gy	0.91
Trachea *D*_max_	1.7 Gy	−0.3 Gy	0.6 Gy	0.1 Gy	0.97
Bronchus *D*_max_	3.9 Gy	0.5 Gy	3.8 Gy	−1.5 Gy	0.96
Great vessels *D*_max_	3.9 Gy	−0.4 Gy	2.9 Gy	−0.4 Gy	0.99
Rib V37.5_Gy_	1.2 cm^3^	0.8 cm^3^	0.2 cm^3^	−0.1 cm^3^	0.93
Lungs‐GTV V12.5_Gy_	104.9 cm^3^	31.2 cm^3^	37.0 cm^3^	−16.6 cm^3^	0.80

	54 Gy in three fractions				
	RMSD (predicted vs. delivered)	Mean difference (predicted ‐ delivered)	RMSD (replanned vs. delivered)	Mean difference (replanned ‐ delivered)	ANOVA *p*‐value
Spinal cord *D*_max_	1.6 Gy	−0.1 Gy	2.1 Gy	−1.0 Gy	0.65
Esophagus *D*_max_	1.9 Gy	0.5 Gy	2.8 Gy	−1.4 Gy	0.36
Heart *D*_max_	2.8 Gy	1.5 Gy	1.3 Gy	−0.1 Gy	0.86
Skin *D*_max_	2.4 Gy	−1.8 Gy	2.0 Gy	−0.9 Gy	0.52
Trachea *D*_max_	2.6 Gy	−1.6 Gy	3.6 Gy	−2.0 Gy	0.65
Rib V30_Gy_	1.4 cm^3^	0.2 cm^3^	1.1 cm^3^	−0.1 cm^3^	0.95
Lungs‐GTV V20_Gy_	1.2%	0.5%	0.4%	0.2%	0.52

**FIGURE 3:**
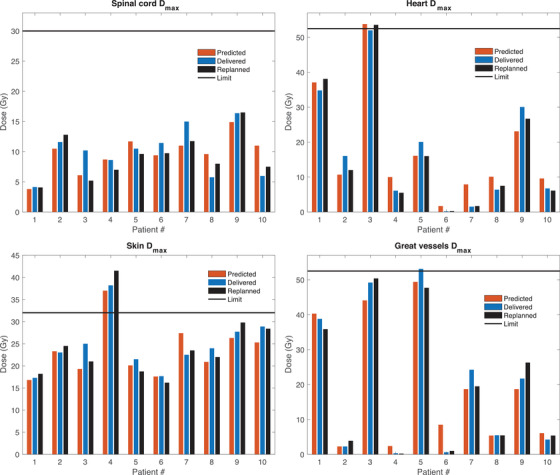
Individual patient dose estimates for the 50 Gy in five fractions protocol comparing the predicted, delivered, and replanned doses for a given organ‐at‐risk (OAR) dose metric. The horizontal black line shows the corresponding OAR dose limit

**FIGURE 4:**
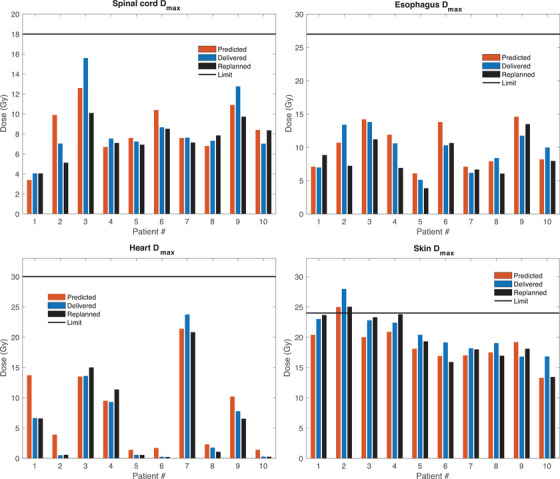
Individual patient dose estimates for the 54 Gy in three fractions protocol comparing the predicted, delivered, and replanned doses for a given organ‐at‐risk (OAR) dose metric. The horizontal black line shows the corresponding OAR dose limit

Figures 3 and 4 show the comparison between predicted, delivered, and replanned dose metrics for each of the individual patients in the test set for the 50 Gy in five fractions and 54 Gy in three fractions protocol, respectively. For the OARs not included in Figures 3 and 4 due to space limitations these data are provided in Figure S1 and Figure S2 in the Supporting Information. Figure 3 shows that the individual dose metrics for the spinal cord, heart, skin, and great vessels generally agreed well between the predicted, delivered, and replanned scenarios. Patients for which the OAR dose metrics were above or close to the tolerance limit were accurately identified as such in the model predictions, such as patients number one, three, and five in terms of the heart and great vessel max dose. Similarly, patients with low OAR doses far from the limit value were predicted as such.

In the case of the 54 Gy in three fractions protocol Figure 4 shows that the dose metrics for the spinal cord, esophagus, heart, and skin agreed well, with the exception of the heart max dose for patient 1. The model correctly identified patients with OAR dose metrics close to or above the tolerance limit also for the 54 Gy in three fractions protocol, although the patients receiving this higher dose protocol had mainly peripheral lung tumors. Furthermore, the spinal cord max dose was considerably lower in the replanned compared to delivered plans for 3/10 patients, and the skin max dose was lower for 4/10 patients.

## 4 DISCUSSION

This study evaluated the performance of ML dose prediction for lung SBRT using a test set of 20 patients, half treated with 50 Gy in five fractions and half with 54 Gy in three fractions. The results showed that the predicted DVH dose metrics agreed well with those from the clinically delivered plans for these patients. There were also several instances where having access to these dose predictions in a blinded re‐plan setting yielded better OAR sparing compared to the clinically delivered plans, despite these plans being generated by junior planners with limited lung SBRT experience. This highlights the potential for improved OAR sparing by using the predicted dose as a further goal to aim for in the treatment planning process, even if the plan already meets the tolerance limit for the respective OAR. This is further supported by a recent study showing that automatically generated treatment plans for lung SBRT were able to achieve lower heart, esophagus, trachea, and spinal cord max doses compared to manual planning.^3^

Generating the dose prediction estimates from the planning CT scan and contours takes approximately ten seconds using the method employed in this study, allowing for a very quick assessment of expected OAR doses and comparison between different fractionation protocols. For patients receiving lung SBRT this can aid the upfront decision‐making process by selecting the optimal fractionation schemes without the need for generating comparative treatment plans. For both fractionation protocols the models accurately captured cases with OAR dose metrics close to the stated tolerance limit, which is key for alerting the planner and treating physician to trade‐offs that need to be addressed.

Similar concepts of DVH prediction have been proposed by others using different methodologies. One research group used two different methods, one based on the uniform field doses of nine equidistant IMRT beams and the other utilizing geometric information about relative target and OAR positions.^10^ With similar results for the two methods the study found mean prediction errors across OARs of between 1% to 8% for nasopharyngeal cancer cases, and 2% to 6% for rectal cancer cases, compared to 0.3% to 3.7% for 50 Gy in five fractions and 0.3% to 7.6% for 54 Gy in three fractions in our study. Another approach also focusing on patients with nasopharyngeal cancer adopted multiple linear fitting of DVH metrics from various sub‐regions of OARs based on how far away each sub‐region is from the target.^12^ This approach showed similar performance with average differences between predicted and delivered mean doses of 1.2% to 4.3% across OARs, albeit with rather large standard deviations (∼5%) indicating that the predictions may not agree well for each individual patient.

There have also been efforts to utilize deep learning methods to predict the entire three dimensions dose distribution rather than focusing on DVH metrics. A recent study used a U‐net implementation to predict the dose distribution for left‐sided breast cancer patients treated with VMAT. This showed promising results when compared with the clinically delivered dose distributions for 10 test cases, with some larger uncertainty seen for the max dose to the lungs and heart.^20^ It is important to note that in addition to average estimates of prediction performance, comparing estimates for individual patients is key to understanding prediction performance, since average doses can agree well even if there are considerable under‐ or overestimates for a given patient.

A limitation of the methodology employed in this study is the need for relatively large training sets to avoid problems with overfitting or not capturing the clinically relevant distribution of anatomical and geometrical variations in target volumes and OARs. Thus, widespread clinical implementation would require large datasets across each disease site, which may be challenging to obtain. While the results presented here show promising prediction performance when evaluated on test patients from our institution, that does not guarantee accurate model performance across other centers where SBRT practices may vary. A strong feature of the current study is the use of multi‐center input data used to construct the models. Generally, a diverse input data set is important when using decision trees, as the learned model would have limited accuracy if extrapolated beyond the input training data. The diversity of the training data used here should capture a large number of clinical scenarios and thereby improve generalizability. Nonetheless, further testing of these models in different institutions with varying SBRT practices is warranted to confirm the results found in our study. To further this point, we would not recommend applying the models developed here to different lung SBRT fractionation schedules without an analysis of proper scaling.

A further enhancement of this type of DVH dose prediction could be to combine it with automated treatment plan generation for example using knowledge‐based techniques. This would allow the potential for generating an individualized automated treatment plan that attempts to achieve the predicted DVH estimates, which can then be improved upon if needed following a quick clinical discussion. Whether used mainly for time‐saving and improving efficiency in treatment plan decision‐making, or to facilitate better OAR sparing and plan consistency, dose prediction is a promising new avenue that can help improve modern radiation oncology practice.

## CONFLICT OF INTEREST

The authors declare no conflicts of interest.

## AUTHOR CONTRIBUTIONS

NPB, LS, CV, JS, AB, NO, MKG, CC, and WAT contributed to the concept and design of the study. WM and SS contributed to data generation and data collection. NPB, CV, NO, and WAT contributed to statistical and data analyses. All authors contributed to the writing or reviewing of the manuscript and have approved the final version.

## Supporting information

SUPPORTING INFORMATION
