# Supplementary material for: Organ‐at‐risk dose prediction using a machine learning algorithm: Clinical validation and treatment planning benefit for lung SBRT
Source: J Appl Clin Med Phys. 2022 Apr 23;23(6):e13609. doi: 10.1002/acm2.13609 (PMC9195027; doi:10.1002/acm2.13609)
Supplement: Supplementary file 1 — SUPPORTING INFORMATION [file ACM2-23-e13609-s001.pdf]

Supplementary material

Supplementary table S1: Machine learning parameters used for the gradient boosting decision tree regression algorithm.

objective	learning_rate	loss	max_depth	min_samples_leaf	min_samples_split	n_estimators
BRONCHUS_V18cc	0.1	lad	10	5	10	300
BRONCHUS_max	0.1	ls	1	2	5	100
CORD_V13.5cc	0.05	huber	5	5	10	300
CORD_V22.5cc	0.1	huber	5	5	5	100
CORD_max	0.05	huber	1	5	5	100
ESOPH_V27.5cc	0.05	ls	5	2	10	300
ESOPH_V60	0.05	ls	5	2	2	100
ESOPH_max	0.05	ls	5	2	2	100
ESOPH_mean	0.05	ls	5	2	10	300
HEART_V32cc	0.05	lad	10	2	10	300
HEART_V50	0.05	lad	5	5	10	300
HEART_max	0.1	lad	10	5	2	100
HEART_mean	0.1	ls	5	5	5	100
LTBRACHPLEX_V30cc	0.05	huber	10	5	5	300
LTBRACHPLEX_max	0.05	lad	5	5	5	300
LTBRACHPLEX_mean	0.05	huber	1	5	2	300
PTVhigh_V45	0.05	lad	10	5	10	100
PTVhigh_V48.6	0.05	lad	1	2	5	300
PTVhigh_V50	0.05	lad	1	5	5	300
PTVhigh_V54	0.05	huber	1	5	5	100
PTVhigh_V60	0.1	lad	1	2	10	100
PTVhigh_min	0.1	lad	10	2	5	100
PTVlow_V45	0.05	lad	5	2	5	300
PTVlow_V48.6	0.1	ls	10	5	2	100
PTVlow_V50	0.1	ls	1	2	10	100
PTVlow_V54	0.1	ls	5	5	5	100
PTVlow_V60	0.1	lad	10	2	2	300
PTVlow_min	0.1	lad	5	5	2	300
PTVprimary_V45	0.05	huber	10	5	10	100
PTVprimary_V48.6	0.1	huber	1	5	2	300
PTVprimary_V50	0.05	huber	1	2	10	300
PTVprimary_V54	0.1	ls	5	2	10	300
PTVprimary_V60	0.05	ls	1	2	5	100

PTVprimary_min	0.05	ls	10	2	10	100
RIB_V30cc	0.1	huber	1	2	10	300
RIB_V37.5cc	0.05	ls	5	2	10	100
RTBRACHPLEX_V30cc	0.1	ls	5	2	10	300
RTBRACHPLEX_max	0.1	lad	5	2	2	300
RTBRACHPLEX_mean	0.1	huber	1	2	5	300
SKIN_V30cc	0.1	ls	5	2	10	100
SKIN_max	0.05	ls	5	2	2	300
TOTLUNGCTV_V20	0.1	lad	10	5	2	300
TOTLUNGCTV_mean	0.1	lad	10	2	10	300
TOTLUNGGTV_V12.5cc	0.1	lad	10	5	5	300
TOTLUNGGTV_V13.5cc	0.05	lad	5	5	10	300
TOTLUNGGTV_V20	0.1	lad	10	2	10	100
TRACHEA_V18cc	0.1	huber	10	2	2	300
TRACHEA_max	0.1	huber	5	5	5	100
VESSELS_V47cc	0.1	huber	1	2	10	100
VESSELS_max	0.05	huber	5	5	5	300

Description of column labels: 'learning_rate' = Learning rate, 'loss' = Loss function for the gradient boosting algorithm, 'max_depth' = Maximum depth of the decision trees, 'min_samples_leaf' = Minimum samples required for a leaf, 'min_samples_split' = Minimum samples to split at a node, 'n_estimators' = Number of trees. Description of loss function labels: 'lad' = Least absolute deviation, 'huber' = Huber loss function, 'ls' = Least squares

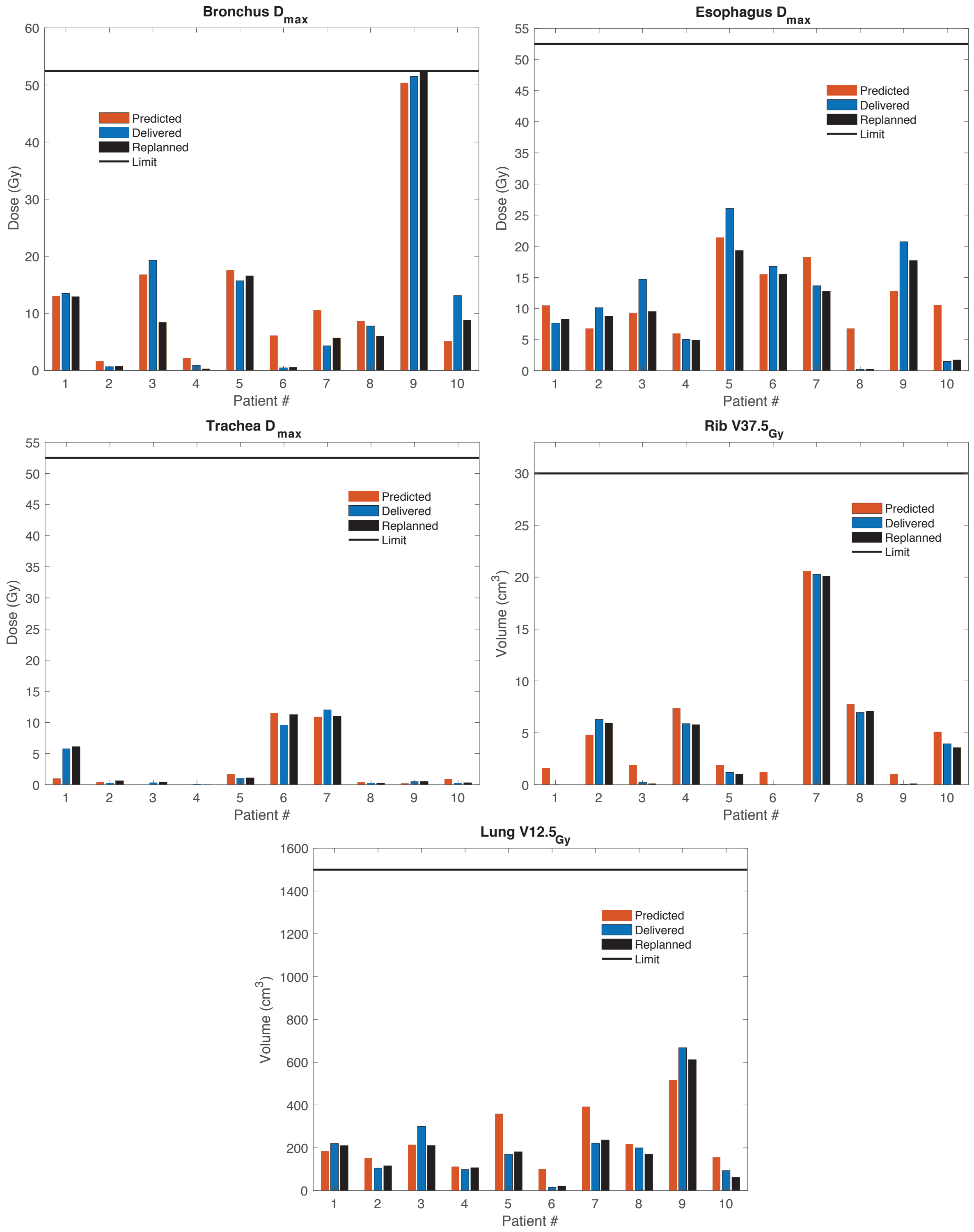

Supplementary figure S1. Individual patient dose estimates for the 50 Gy in 5 fractions protocol comparing the predicted, delivered and replanned doses for a given organ-at-risk (OAR) dose metric. The horizontal black line shows the corresponding OAR dose limit.

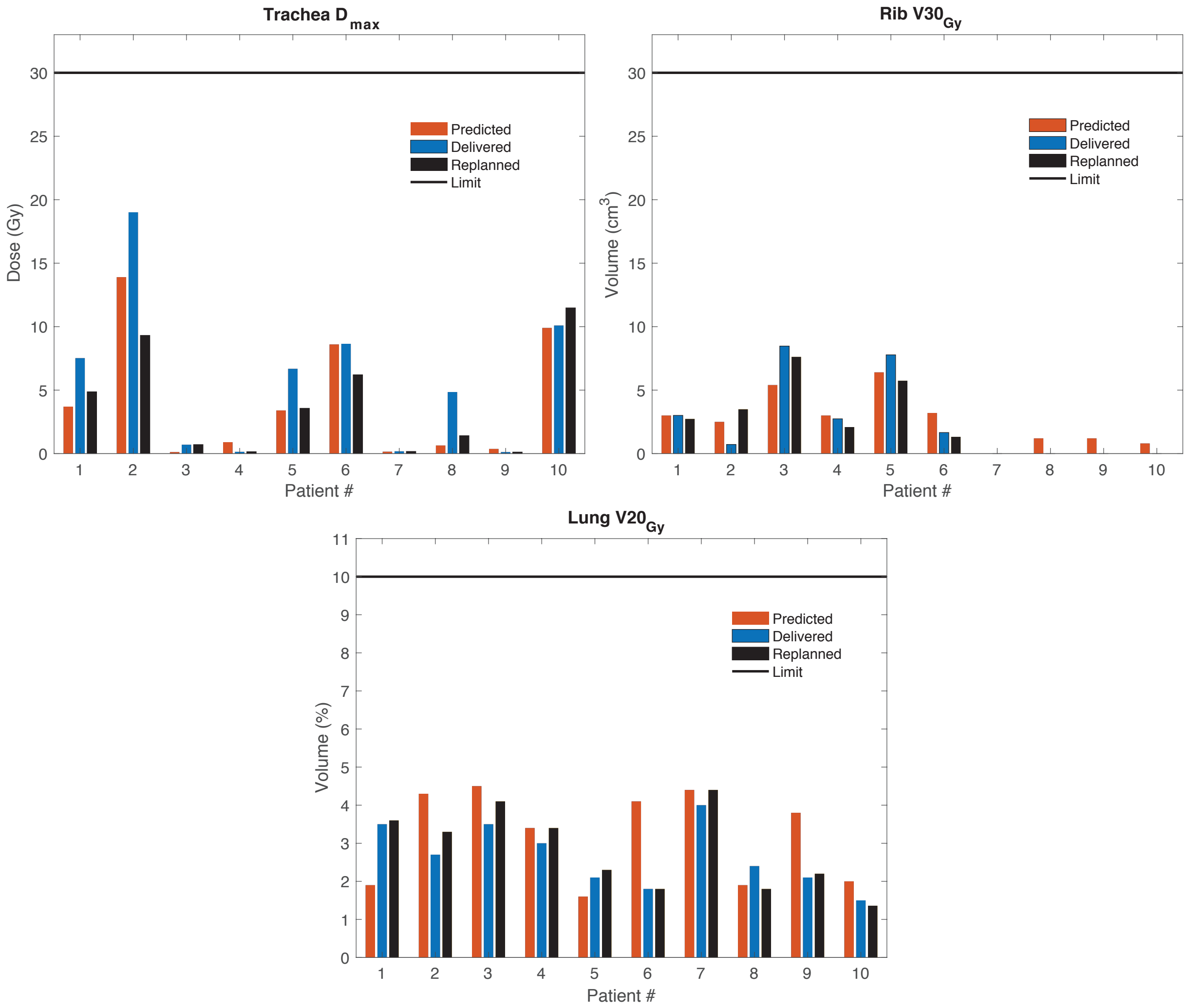

Supplementary figure S2. Individual patient dose estimates for the 54 Gy in 3 fractions protocol comparing the predicted, delivered and replanned doses for a given organ-at-risk (OAR) dose metric. The horizontal black line shows the corresponding OAR dose limit.
